# Supplementary material for: Mucosal absorption of therapeutic peptides by harnessing the endogenous sorting of glycosphingolipids
Source: eLife. 2018 May 31;7:e34469. doi: 10.7554/eLife.34469 (PMC5980230; doi:10.7554/eLife.34469)
Supplement: Transparent reporting form [file elife-34469-transrepform.docx]

***eLife’s* transparent reporting form**

We encourage authors to provide detailed information *within their submission* to facilitate the interpretation and replication of experiments. Authors can upload supporting documentation to indicate the use of appropriate reporting guidelines for health-related research (see [EQUATOR Network](http://www.equator-network.org/%20)), life science research (see the [BioSharing Information Resource](https://biosharing.org/)), or the [ARRIVE guidelines](http://www.plosbiology.org/article/info:doi/10.1371/journal.pbio.1000412) for reporting work involving animal research. Where applicable, authors should refer to any relevant reporting standards documents in this form.

If you have any questions, please consult our Journal Policies and/or contact us: [editorial@elifesciences.org](mailto:editorial@elifesciences.org).

**Sample-size estimation**

- You should state whether an appropriate sample size was computed when the study was being designed
- You should state the statistical method of sample size computation and any required assumptions
- If no explicit power analysis was used, you should describe how you decided what sample (replicate) size (number) to use

Please outline where this information can be found within the submission (e.g., sections or figure legends), or explain why this information doesn’t apply to your submission:

We did not use an explicit power analysis to calculate sample size. At the outset, we began using 3 mice per group in blood absorption experiments, which was sufficient sample size to show statistical significance. To show reproducibility, replicate studies included groups with 2-5 mice.

All raw data for GTT studies (total area under glucose tolerance curves; AUC) are explicitly shown in Figure 5C and 5D. Other *in vivo* experiments use standard curves to quantify absorption as described in Material and Methods.

Source Data used to produce figures for in vivo experiments (Figures 4A, 4E, 5D, 5E, and 5F) are provided.

**Replicates**

- You should report how often each experiment was performed
- You should include a definition of biological versus technical replication
- The data obtained should be provided and sufficient information should be provided to indicate the number of independent biological and/or technical replicates
- If you encountered any outliers, you should describe how these were handled
- Criteria for exclusion/inclusion of data should be clearly stated
- High-throughput sequence data should be uploaded before submission, with a private link for reviewers provided (these are available from both GEO and ArrayExpress)

Please outline where this information can be found within the submission (e.g., sections or figure legends), or explain why this information doesn’t apply to your submission:

See individual figure legends for number of independent studies and number of replicates. In brief, each measurement was an average of 2 technical replicates and each condition included at least 2 biological replicates. Each *in vivo* experiment was reproduced at least 2 independent times. Oral blood absorption experiments were reproduced in 5 independent experiments (Figure 4A). *In vivo* GTT experiments were reproduced 8 times in 2 different laboratories (and animal housing facilities) (Lencer and Khan).

All outliers are included in all analysis for all figures except for Figure 5E. In Figure 5E, experiment #3 (shown in Source Data File) was excluded from analysis because the standard curve was not reliable. Including these data does not change result. Raw data are submitted for peer review for in vivo experiments shown in Figures 4A, 4E, 5D, 5F, and including 5E.

**Statistical reporting**

- Statistical analysis methods should be described and justified
- Raw data should be presented in figures whenever informative to do so (typically when N per group is less than 10)
- For each experiment, you should identify the statistical tests used, exact values of N, definitions of center, methods of multiple test correction, and dispersion and precision measures (e.g., mean, median, SD, SEM, confidence intervals; and, for the major substantive results, a measure of effect size (e.g., Pearson's r, Cohen's d)
- Report exact p-values wherever possible alongside the summary statistics and 95% confidence intervals. These should be reported for all key questions and not only when the p-value is less than 0.05.

Please outline where this information can be found within the submission (e.g., sections or figure legends), or explain why this information doesn’t apply to your submission:

See individual figure legends for description of statistics used. In general, ANOVA and multiple comparisons were performed using Graph Pad PRISM software.

(For large datasets, or papers with a very large number of statistical tests, you may upload a single table file with tests, Ns, etc., with reference to sections in the manuscript.)

**Group allocation**

- Indicate how samples were allocated into experimental groups (in the case of clinical studies, please specify allocation to treatment method); if randomization was used, please also state if restricted randomization was applied
- Indicate if masking was used during group allocation, data collection and/or data analysis

Please outline where this information can be found within the submission (e.g., sections or figure legends), or explain why this information doesn’t apply to your submission:

Mice in each individual experiment were from same lot (Jackson Laboratories).

**Additional data files (“source data”)**

- We encourage you to upload relevant additional data files, such as numerical data that are represented as a graph in a figure, or as a summary table
- Where provided, these should be in the most useful format, and they can be uploaded as “Source data” files linked to a main figure or table
- Include model definition files including the full list of parameters used
- Include code used for data analysis (e.g., R, MatLab)
- Avoid stating that data files are “available upon request”

Please indicate the figures or tables for which source data files have been provided:

Source data used to produce Figures 4A, 4E, 5D, 5E, and 5F are provided as individual excel files.
